# Estimating high-density aboveground biomass within a complex tropical grassland using Worldview-3 imagery

**DOI:** 10.1007/s10661-024-12476-7

**Published:** 2024-03-15

**Authors:** Rowan Naicker, Onisimo Mutanga, Kabir Peerbhay, Omosalewa Odebiri

**Affiliations:** 1 Discipline of Geography, School of Agricultural, Earth and Environmental Sciences, University of KwaZulu-Natal, Private Bag X01, Scottsville, Pietermaritzburg, 3209 South Africa; 2 Centre for Integrative Ecology, School of Life and Environmental Sciences, Deakin University, VIC 3125 Melbourne, Australia

**Keywords:** Aboveground biomass, Biomass saturation, Linear stretch, Histogram equalization, Normalized difference vegetation index, Random forest, Worldview-3

## Abstract

A large percentage of native grassland ecosystems have been severely degraded as a result of urbanization and intensive commercial agriculture. Extensive nitrogen-based fertilization regimes are widely used to rehabilitate and boost productivity in these grasslands. As a result, modern management frameworks rely heavily on detailed and accurate information on vegetation condition to monitor the success of these interventions. However, in high-density environments, biomass signal saturation has hampered detailed monitoring of rangeland condition. This issue stems from traditional broad-band vegetation indices (such as NDVI) responding to high levels of photosynthetically active radiation (PAR) absorption by leaf chlorophyll, which affects leaf area index (LAI) sensitivity within densely vegetative regions. Whilst alternate hyperspectral solutions may alleviate the problem to a certain degree, they are often too costly and not readily available within developing regions. To this end, this study evaluated the use of high-resolution Worldview-3 imagery in combination with modified NDVI indices and image manipulation techniques in reducing the effects of biomass signal saturation within a complex tropical grassland. Using the random forest algorithm, several modified NDVI-type indices were developed from all potential dual-band combinations of the Worldview-3 image. Thereafter, linear contrast stretching and histogram equalization were implemented in conjunction with Singular Value Decomposition (SVD) to improve high-density biomass estimation. Results demonstrated that both contrast enhancement techniques, when combined with SVD, improved high-density biomass estimation. However, linear contrast stretching, SVD, and modified NDVI indices developed from the red (630–690 nm), green (510–580 nm), and near-infrared 1 (770–895 nm) bands were found to produce the best biomass predictive model (*R*^2^ = 0.71, RMSE = 0.40 kg/m^2^). The results generated from this research offer a means to alleviate the biomass saturation problem. This framework provides a platform to assist rangeland managers in regionally assessing changes in vegetation condition within high-density grasslands.

## Introduction

Grasslands are dynamic environments that sustain vital ecosystem goods and services, such as climate regulation and carbon sequestration (Egoh et al., 2011; Hanski, 1999; Naicker et al., 2016). Despite this, expedited urbanization and intensified agriculture have severely degraded many native grasslands. To rehabilitate these degraded ecosystems, rangeland management techniques often utilize extensive nitrogen-based fertilization regimes, which are designed to drastically bolster rangeland productivity (Muir et al., 2001; Omaliko et al., 1984). To ascertain rangeland condition, aboveground biomass (AGB) productivity has often been utilized as a measure of rangeland health and stability (Psomas et al., 2011; A. Ramoelo et al., 2015a, 2015b). Consequently, the accurate estimation of AGB provides the detailed information required to regionally assess and monitor the health and stability of grassland ecosystems (Sibanda et al., 2017).

The advent of optical remote sensing has superseded conventional laboratory-based techniques as the most efficient means to derive information on grassland condition (Asner, 1998; Ramoelo et al., 2012; Xie et al., 2009). Hyperspectral remote sensing, which comprises of numerous contiguous bands, has been extensively used to estimate grassland AGB (Ling et al., 2014; Mutanga, 2004; Mutanga et al., 2012; Psomas et al., 2011). However, due to its exorbitant cost, high degree of dimensionality, and lack of availability within developing regions, its practical use has often been limited (Ramoelo et al., 2015a, 2015b; Sibanda et al., 2015a, 2015b). Nevertheless, modern multispectral sensors (e.g., Worldview, Landsat OLI, Sentinel-2) with improved spectral and spatial capabilities have presented a viable alternative (Sibanda et al., 2015a, 2015b). Several studies have proven the use of these sensors in mapping AGB (Mutanga et al., 2012; Ramoelo et al., 2015a, 2015b; Shoko et al., 2018; Sibanda et al., 2017). For example, Mutanga et al. (2012) used Worldview-2 (400–1040 nm) imagery, modified band ratios, and random forest models to successfully estimate wetland biomass (*R*^2^ = 0.79) along the eastern coast of Southern Africa. Whilst Shoko et al. (2018) applied Sentinel-2 (442–2190 nm) imagery and an advanced sparse partial least squares regression to map AGB for C3 and C4 grass species (*R*^2^ = 0.84) within the Drakensberg region of South Africa. Lastly, Sibanda et al. (2017) successfully employed sparse partial least squares regression and texture-based Worldview-3 (400–2365 nm) spectral derivatives to estimate AGB of tropical grasses (*R*^2^ = 0.90) within KZN, South Africa. The reported successes have predominately been realized through the use of vegetation indices, such as the normalized difference vegetation index (NDVI) (Karlson et al., 2015; Lu, 2006; Mutanga et al., 2012).

Be that as it may, the use of standard broad-band vegetation indices is often subjected to the problem of biomass signal saturation in dense vegetative regions (Mutanga & Skidmore, 2004; Mutanga et al., 2012). Biomass signal saturation is a renowned challenge that has long affected the remote sensing community (Mutanga & Skidmore, 2004; Mutanga et al., 2012). Mutanga et al. (2012), in particular, noted that NDVI calculated from broad-band sensors typically reaches signal saturation in regions where AGB is equivalent to ± 0.3 g/cm^2^. Intrinsically, as documented by Gitelson et al. (1996), Mutanga and Skidmore (2004), and Mutanga et al. (2012), in instances of high biomass concentrations, a strong chlorophyll absorption band located within the red section of the electromagnetic spectrum rapidly saturates (Gitelson et al., 1996). This occurrence renders broad-band indices developed from the standard near-infrared and red bands (such as NDVI) prone to biomass signal saturation (Gitelson et al., 1996; Mutanga & Skidmore, 2004). In response, Mutanga and Skidmore (2004) proposed the use of narrow-band vegetation indices to combat this problem. They tested the use of three indices (modified NDVI, simple ratio, and the transformed vegetation index) to estimate pasture biomass. Their results indicated that the use of narrow-band indices drastically improved biomass estimates, with *R*^2^ values increasing from 0.26 to 0.77. Although encouraging, the use of hyperspectral data comes with its own potential drawbacks (as earlier highlighted) which in turn limits its regional practical application (Ramoelo et al., 2015a, 2015b; Sibanda et al., 2015a, 2015b). Considering this, in a later study, Mutanga et al. (2012) tested the use of Worldview-2 imagery and modified NDVI indices in estimating high-density AGB within a South African wetland. Their results showed that *R*^2^ values improved from 0.39 to 0.79 with the use of modified NDVI indices. This outcome demonstrates the potential feasibility of broad-band modified NDVI indices in estimating high-density grassland biomass.

Despite improvements in sensor capabilities, limited work has been done to further improve high-density AGB estimation within grasslands, with no standardized approach currently adopted. The lack of a standardized approach is particularly problematic in instances of high fertilization within poorly managed rangelands, where the assessment of subtle deviations in grassland biomass is required to monitor productivity and deter further degradation. In this regard, an alternate solution to the biomass saturation problem may lie within the further examination of both the saturation issue and the nature of the NDVI equation. Rouse et al. (1974) first introduced the NDVI numerical transformation as an indicator of vegetation condition and to counteract the effects of background interferences. The equation is fundamentally a non-linear mathematical stretch of the near-infrared/red band ratio and is designed to constrain its values from − 1 to + 1 (Huete et al., 1999; Mróz & Sobieraj, 2004; Rouse et al. 1974). NDVI values for vegetated regions generally range between 0.1 and 0.7, with values larger than 0.5 indicating densely vegetated areas (Rulinda et al., 2012). However, due to the specific bands used in the NDVI equation (i.e., red and NIR), the saturation effect arises when the difference in absorption between the two bands is very large, such as in instances of high leaf area index (LAI), which is commonly associated with regions of high vegetation density. This outcome leads to a limited ability to detect variations in densely vegetated regions, as noted by Huete et al. (1999). In these regions, the transparency of plant leaves in near-infrared (NIR) light contrasts with their high absorption of red wavelengths (Tucker, 1977). Thus, as the LAI increases, the change in NIR reflectance is comparatively minor, whereas red reflectance experiences a rapid decline (Mutanga & Skidmore, 2004). This phenomenon, however, can be less pronounced with the use of Red-edge-based vegetation indices, due to the narrower differences between NIR and Red-edge wavelengths as well as their penetrative capacity (Mutanga & Skidmore, 2004). Nevertheless, the inclusion of a linear mathematical stretch — comprised of a linear algebraic stretch and an image enhancement feature — prior to the development of an NDVI vegetation index may provide a greater range for previously constrained higher biomass values and effectively reduce the biomass saturation effect (Huete et al., 1999).

Contrast enhancement techniques have been used extensively across disciplines to facilitate image analysis (Buka et al., 2017; Demirel et al., 2009; Grundland & Dodgson, 2006). Linear contrast stretching, which reallocates the original digital values to increase the dynamic range of gray levels within an image (Al-amri et al., 2010), and histogram equalization, which redistributes all pixel values within an image to generate a uniformly distributed histogram (Osman et al., 2009), are the most widely implemented image enhancement techniques (Chang & Wu, 1998; Kim et al., 2001). For instance, both Lobo et al. (1998) and Buka et al. (2017) utilized contrast enhancement techniques to facilitate grassland and forest mapping assessments. However, although useful, contrast enhancement techniques provide a mere step within the overall image manipulation procedure, by accentuating visual characteristics within the image prior to mathematical manipulation (Demirel et al., 2009). Consequently, a mathematical tool, which can calculate not only the eigenvalues but also eigenvectors within an image, is an imperative next step (Susanto et al., 1998). In this regard, Singular Value Decomposition (SVD), which provides a means to factorize a matrix into singular values and vectors, is a renowned technique within linear algebra — owing to its ability to transform a digital image into three specific matrices (Cao, 2006; Demirel et al., 2009). Although, SVD has had several applications within statistics, machine learning, and computer science, SVD has principally only been applied for classification-based studies within remote sensing (Ball et al., 2004; Danaher et al., 1995; Lisowski & Cook, 1996; Phillips et al., 2009). For instance, Danaher et al. (1995) utilized SVD and key vector analysis to successfully classify forest species from multispectral data, whilst Ball et al. (2004) used SVD to facilitate hyperspectral pixel unmixing.

Nevertheless, regardless of the simple and expedient numerical characteristics associated with SVD, to the best of our knowledge, few remote sensing researchers have incorporated it within prediction-based investigations. For example, Marshall and Thenkabail (2015) utilized SVD prior to vegetation index development to facilitate crop biomass estimation. Using multiband vegetation indices developed from SVD and stepwise regressions, they successfully obtained biomass estimation models for rice (*R*^2^ = 0.91), alfalfa (*R*^2^ = 0.81), cotton (*R*^2^ = 0.97), and maize (*R*^2^ = 0.94). In a later study, Ji and Fan (2019) successfully coupled SVD analysis, Eurasian NDVI, and climate factors to develop Eurasian climate prediction models (*R*^2^ = 0.92, RMSE = 0.04). Consequently, given the hindrance posed by biomass signal saturation within densely vegetated rangelands, and the limited use of SVD within estimation-based remote sensing studies, this study set out to develop an applied framework to combat the effects of biomass signal saturation within a heterogeneous tropical grassland environment. Thus, this investigation evaluated the use of high-resolution Worldview-3 imagery in combination with modified band ratio indices and an image manipulation procedure (which incorporated the use of contrast enhancement techniques and Singular Value Decomposition) to mitigate the influence of biomass signal saturation within densely vegetated grassland plots.

## Materials and methods

### Study area

**Fig. 1:**
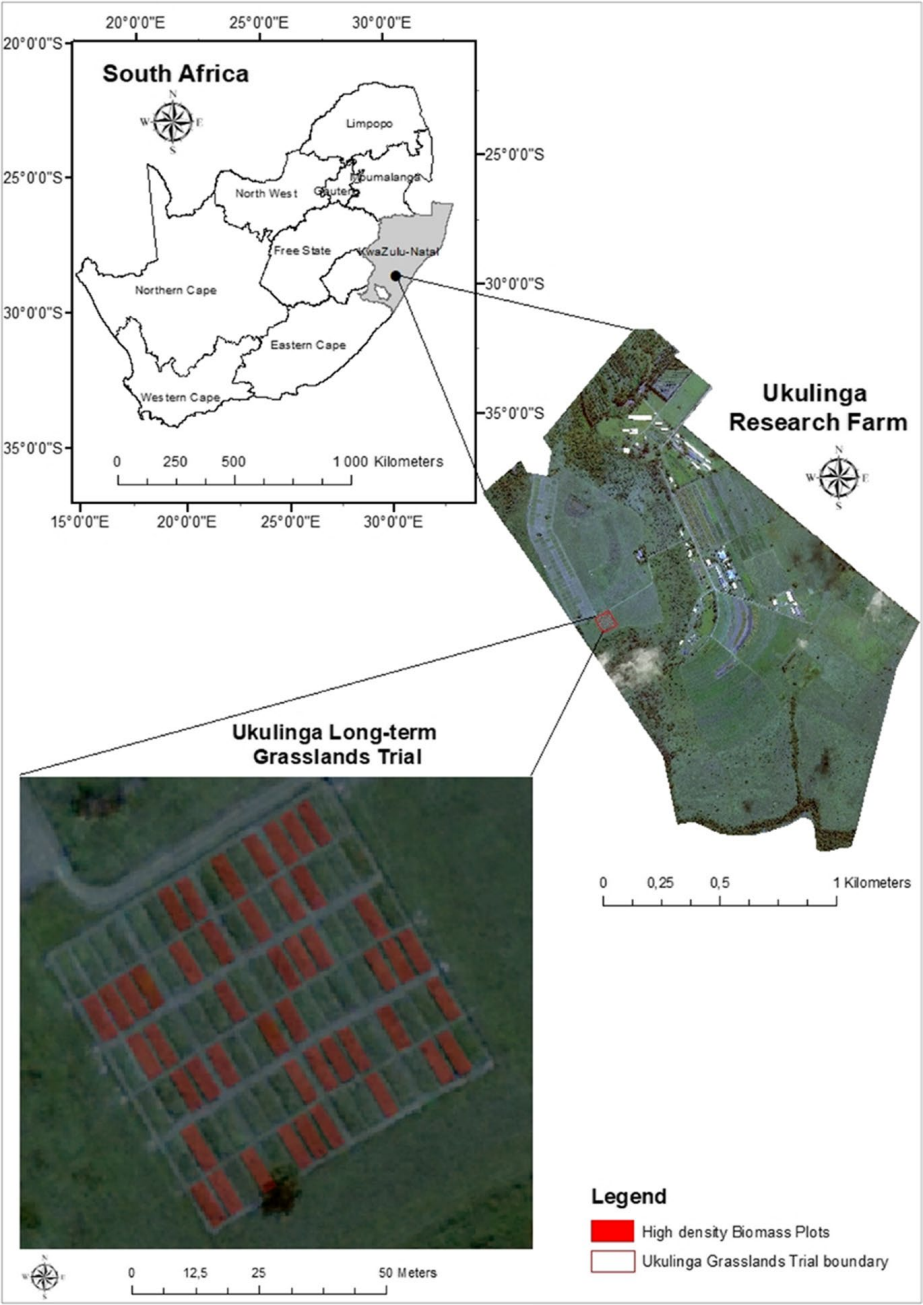
Location of the high-density biomass plots within the Ukulinga study site, represented on an original true color Worldview-3 8-band image with and RGB band combination

A grassland fertilization trial, within the Ukulinga research farm, was selected as the study area for the investigation (Fig. 1). Several grass species, namely, *Themeda triandra* (Red grass), *Heteropogon contortus* (Black speargrass), *Eragrostis plana* (Cane grass), *Panicum maximum* (Guinea grass), *Setaria nigrirostris* (Black-seed bristle grass), and *Tristachya leucothrix* (Trident grass), grow on acidic and infertile Westleigh form soils along the site (Fynn & O'connor, 2005; Morris & Fynn, 2001). Annual rainfall (694 mm) predominately occurs during the grass growing period of October to April (Sibanda et al., 2015a, 2015b). The fertilization treatment trial itself comprised of 96 experimental plots, each 9 m × 3 m in dimension. Two types of ammonium fertilizers, namely, ammonium nitrate (NH_4_NO_3_) and ammonium sulfate ((NH_4_)_2_SO_4_), were randomly assigned within three replicate blocks (Sibanda et al., 2017). These fertilizers were applied at three distinct levels twice per annum. NH_4_NO_3_ was applied at 21.0 g/m^2^, 42.1 g/m^2^, and 63.2 g/m^2^, whilst (NH_4_)_2_SO_4_ was applied at 33.6 g/m^2^, 67.2 g/m^2^, and 100.8 g/m^2^ (Fynn & O'connor, 2005). These distinct fertilization levels facilitated the characterization of low (0–30%) and high (40–80%) nitrogen fertilization plots (Amanullah et al., 2009; Isleib, 2017; Li et al., 2018), which were later used to assist in the identification of high-density biomass plots (where AGB ≥ 3 kg/m^2^) using dry weight.

### Field data acquisition

To obtain AGB and distinguish highly dense biomass plots (where AGB ≥ 3 kg/m^2^) (Mutanga et al., 2012), 10 grass samples were collected for each of the 96 experimental plots (each 9 m × 3 m in dimension) to achieve a representative data collection corresponding to the 2 m spatial resolution of the Worldview-3 sensor. This representative data collection was conducted to ensure a meaningful spatial coverage relative to the sensor’s capabilities, thereby ensuring precise biomass estimation through an appropriate encapsulation of the landscape’s heterogeneity. Wet grass samples from each plot were cut and collected at peak grass growth (April 2017) and stored in sealed plastic bags (Ramoelo et al., 2015a, 2015b). These samples were weighed, and then dried within a laboratory oven for a period of 48 h at 70 °C, prior to being reweighed (Sibanda et al., 2015a, 2015b). These readings were then transformed to acquire the total AGB for each plot in kilograms per plot (kg/plot) (Abel Ramoelo et al., 2015a, 2015b; Sibanda et al., 2015a, 2015b). Following these computations, 47 plots corresponding to high nitrogen fertilization treatments (40–80%) were identified as high-density biomass plots (where AGB ≥ 3 kg/m^2^) suitable for the investigation (Fig. 1).

### Image acquisition and pre-processing

The 2 m Worldview-3 image (400–1040 nm) was obtained from the supplier, Swift Geospatial, on the 2nd of May 2017 under cloudless conditions and within 1 week of field sampling. The spectral range of the eight Worldview-3 wavebands are as follows: 400–450 nm (Band 1—coastal); 450–510 nm (Band 2—blue); 510–580 nm (Band 3—green); 585–625 nm (Band 4—yellow); 630–690 nm (Band 5—red); 705–745 nm (Band 6—Red-edge); 770–895 nm (Band 7—near-infrared 1); and 860–1040 nm (Band 8—near-infrared 2). The image was both orthorectified (± 3 m CE90 relative accuracy) and atmospherically corrected by Swift Geospatial. Subsequently, using both field data and GPS readings, a point map for the grasslands trial was generated and overlaid onto the Worldview-3 image. This point map was then used to ascertain the overall image accuracy (89%) and aid in the creation of a map (Fig. 1) detailing the plots of interest (*n* = 47). Lastly, with the use of the plots of interest map, spectral information from each of the wavebands were extracted using the zonal statistics feature in ArcGIS 10 (ESRI, 2011). Due to the homogenous, single land-use nature of the study site, a supervised classification was not applied prior to the extraction of grassland spectral information.

### Image enhancement

**Fig. 2:**
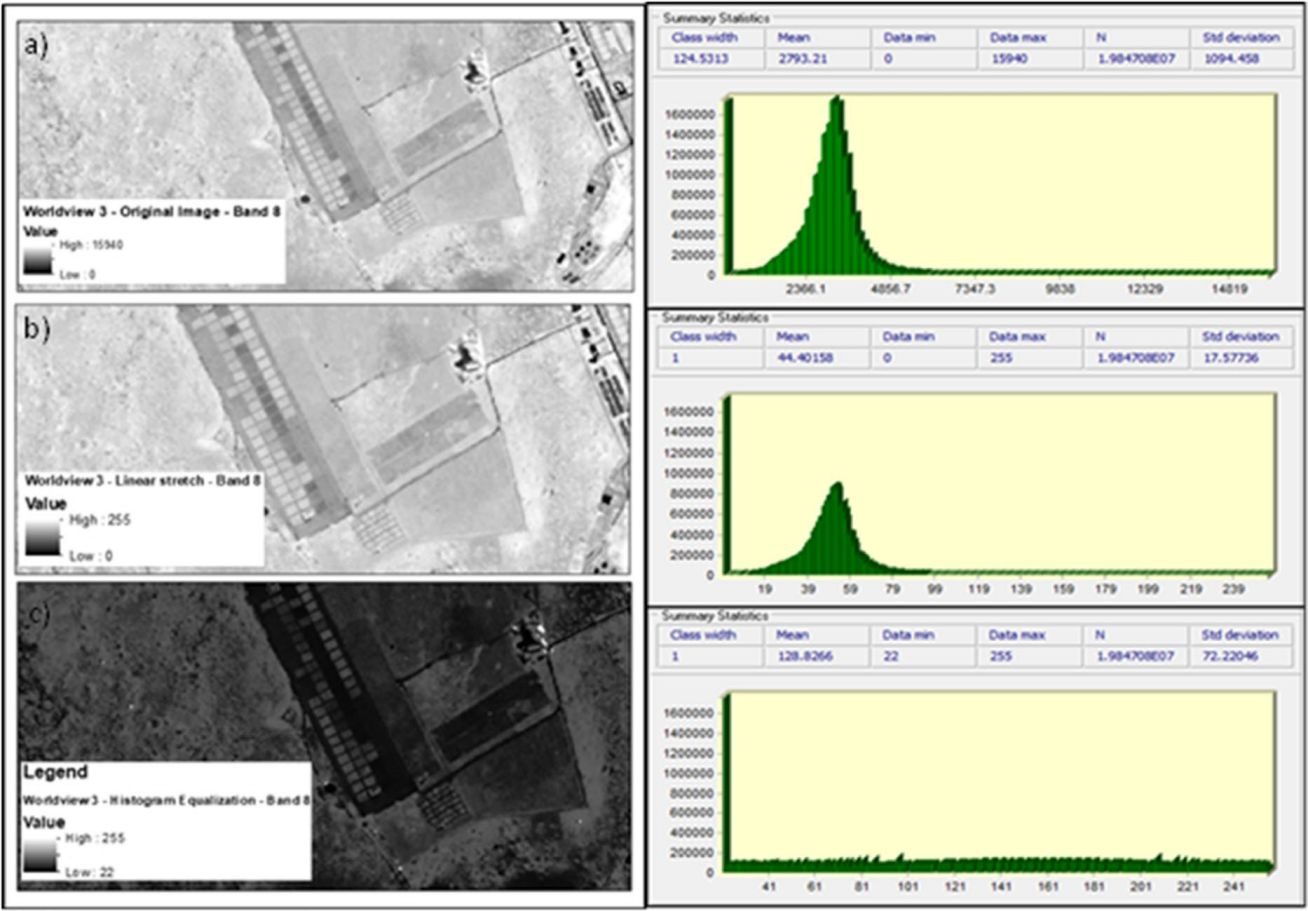
Example of pixel distribution and image contrast before and after image enhancement; **a** a single band (Band 8—NIR 2) from original 8-band Worldview-3 image, **b** the same band which has been linearly stretched (min = 0, max = 255), and **c** the same band which has undergone histogram equalization (min = 0, max = 255)

Two image enhancement techniques were implemented in combination with Singular Value Decomposition (SVD) to facilitate biomass estimation within the high-density plots. Firstly, a min–max linear contrast stretch was applied separately to each of the Worldview-3 wavebands. This method linearly reassigns the initial minimum and maximum values of the image to a new specific set of available pixel values (Chang & Wu, 1998; Grundland & Dodgson, 2006). This was performed to improve both the contrast and brightness of the image prior to the application of SVD (Al-amri et al., 2010). The newly stretched image enabled greater discernment of more subtle variations within the image data by facilitating matrix decomposition (Demirel et al., 2009). Secondly, a histogram equalization stretch was employed. Histogram equalization is a widely applied method owing to its ease and efficacy (Asner, 1998; Demirel et al., 2009). This method reassigns all pixel values equally across the user-defined output classes (Abdullah-Al-Wadud et al., 2007). This change increases the image contrast by stretching the dynamic range of important objects whilst compressing and reducing the contrast of less important objects within the histogram’s array (Al-amri et al., 2010; Kim et al., 2001). The histogram equalization method, however, can be limited by its inability to accommodate disparities in regional brightness (Osman et al., 2009). Nevertheless, this technique has been used extensively within remote sensing and other disciplines as it provides a suitable balance between image enhancement and distortion (Abdullah-Al-Wadud et al., 2007; Al-amri et al., 2010; Demirel et al., 2009). Thereafter, utilizing a combination of the ERDAS imagine, and Idrisi Terrset software’s, both a linear contrast and a histogram equalization stretch were separately applied to each of the Worldview-3 wavebands (Eastman, 2014; Geosystems, 2004). New minimum and maximum pixel values for each image were reassigned within the range of 0–255 (Liu et al., 2012). These image enhancement techniques, which improved image intensity information by accentuating dark and light objects within the image, facilitated feature identification prior to SVD feature extraction (Bhandari et al., 2012; Demirel et al., 2009). Figure 2 shows the changes to the original image after the application of both contrast enhancement techniques. Following image enhancement, single value decomposition was applied within the R statistical environment (Team, 2016).

### Singular Value Decomposition

Singular Value Decomposition (SVD) is a matrix factorization procedure that is more straightforward to implement and has superior mathematical properties than the widely used principal component analysis (Cao, 2006; Marshall & Thenkabail, 2015). SVD is commonly used to reduce a matrix to its simplistic components in order to facilitate matrix calculations (Cao, 2006; Danaher et al., 1995). Subsequently, datasets with a greater number of features than observations can be reduced to a smaller subset of features most prudent to prediction analyses (Bekara & Van der Baan, 2007; Danaher et al., 1995). Moreover, SVD can be particularly useful in instances where coherent events within a dataset can be aligned laterally (Cao, 2006; Marshall & Thenkabail, 2015), such as in instances of biomass signal saturation. Thus, SVD can be applied to improve the signal-to-noise ratio within data segments comprising of laterally coherent events (Bekara & Van der Baan, 2007), like high-density biomass signal saturation. This is carried out by factoring in the greatest singular value contributions (which signify the laterally coherent signals), whilst the lowest singular values are associated with the background noise (Bekara & Van der Baan, 2007). Consequently, SVD can either be implemented as a separate method or as a preliminary stage within a larger framework. The formula used to compute Singular Value Decomposition is detailed in Eq. (1) below:

1 $$M=U\sum {V}^{*}$$

where *M* is a *m* × *m* unitary matrix, *U* is a *n* × *n* unitary matrix (left singular vectors), *Σ* is a *m* × *n* diagonal matrix with non-negative real numbers, *n* represents the number of columns in the original matrix, and V* is a diagonal matrix where the number of elements is less than *M* or *N* (Marshall & Thenkabail, 2015).

Within this study, *M* consisted of the Worldview-3 spectral bands and AGB samples (Marshall & Thenkabail, 2015). The dimensions of the left singular vectors (*U*) equaled the number of Worldview-3 spectral band predictors of the right singular vectors, and (*V*) equaled the number of AGB samples. The decomposition, therefore, produced *N* linear combinations, whose loadings described the relative strength of the predictors on each component (Marshall & Thenkabail, 2015). A typical 0.5 threshold value was applied (Danaher et al., 1995). The Singular Value Decomposition calculation was performed for both contrast enhancement techniques within the R statistical software package, using the ‘‘SVD” function (Team, 2016).

### Vegetation indices

Several different vegetation indices were developed to assist in diminishing the effects of biomass signal saturation and to evaluate the use of Worldview-3 imagery and image manipulation techniques (*n* = 84). Initially, NDVI-based vegetation indices were developed from all potential two-band combinations of the Worldview-3 image (Mutanga et al., 2012). However, due to the unique spectral configuration of the Worldview-3 sensor, a combination of near-infrared 1 and near-infrared 2 (hereafter referred to as NIR3) was also considered (*n* = 69). The formula used to compute each of the discrete modified band combinations is detailed in Eq. (2) below:

2 $${\text{NDVI}}=\frac{{R}_{\left({\text{i}},{\text{n}}\right)}- {R}_{\left({\text{j}},{\text{n}}\right)}}{{R}_{\left({\text{i}},{\text{n}}\right)}+ {R}_{\left({\text{j}},{\text{n}}\right)}}$$

where *R*_(i,n)_ and *R*_(j,n)_ represent the reflectance from any two spectral band combinations of the Worldview-3 image (Mutanga et al., 2012) (see appendix 1 for a full description of all band combinations used). Nevertheless, to further increase the accuracy of high-density AGB estimation, several other indices, namely, simple ratio, enhanced vegetation index, soil-adjusted vegetation index, and the green difference vegetation index, were used (see appendix 2) (Jurgens, 1997; Mutanga et al., 2012; Mutanga & Skidmore, 2004; Ramoelo et al., 2015a, 2015b). The performance of the Worldview-3 spectral bands, vegetation indices, and the combination of the two in estimating high-density biomass were subsequently evaluated. Lastly, each of these variables was separately input into the random forest regression algorithm to ascertain the ability of each in estimating high-density AGB.

### Random forest regression

The random forest algorithm is an ensemble-based technique that utilizes a large set of decision trees to contend with regression-based tasks (Breiman, 2001). The benefit of the random forest method lies within its ability to cope with both highly correlated and noisy predictor variables (Lin et al., 2010). In order to build each tree within the random forest decision tree matrix, a deterministic algorithm selects several bootstrapped samples, which are then drawn with replacements (Mutanga et al., 2012; Peerbhay et al., 2016). Subsequently, all trees are then grown to a user-defined node size, where the final prediction is ascertained by averaging each specific tree prediction (Adam et al., 2014; Breiman, 2001). The random forest regression algorithm was implemented within the R statistical software package (Team, 2016).

For the development of the high-density biomass models, the dataset was split into 70% training (*n* = 32) and 30% testing (*n* = 15) data (Peerbhay et al., 2016). Thereafter, specific user parameters had to be set within the RF model (Breiman, 2001). To begin with, the number of decision trees to be drawn with replacements (*ntree* = 1000) had to be defined. Next, the number of predictor variables (*mtry*) to be tested at each node was determined (Lin et al., 2010). The *mtry* values were derived through the default value of the square root of the total number of predictor variables (spectral variables) used (Ramoelo et al., 2015a, 2015b). To improve the model’s accuracy, both *ntree* and *mtry* values were optimized, and the model was run 100 times (Mutanga et al., 2012). Out of the bag (OOB) data that was not included within the bootstrapped sample was then employed to estimate the importance of the predictor variables (Karlson et al., 2015). The most important variables were identified using a backwards feature elimination method (which eliminates the least useful variable within the model (Karlson et al., 2015). In addition, using both the original and manipulated Worldview-3 images, several predictive models for each discrete modified NDVI index, traditional vegetation index, and Worldview-3 waveband were created. Thereafter, with the use of the three most important predictor variables, an optimized model was produced (Mutanga et al., 2012). Finally, a tenfold cross-validation approach was implemented to assess the predictive performance of each biomass model (Karlson et al., 2015; Mutanga et al., 2012). The standard coefficient of determination (*R*^2^) (which ranges from 0 to 1, with 1 indicating the proportion of dataset variance explained by the model) and the root mean square error (RMSE) (where the RMSE summarizes the prediction error in the units of the original measurements, here a lower RMSE is preferred) were used (Adam et al., 2014).

## Results

### Descriptive statistics of grassland biomass

High biomass was observed predominately within the grassland plots fertilized by greater quantities of inorganic nitrogen (high nitrogen plots). The average biomass recorded within the high-density plots was 3.37 kg/m^2^ (Table 1). This is similar to the standard level of biomass density required to induce biomass signal saturation, as described by both Mutanga and Skidmore (2004) and Mutanga et al. (2012).

**Table 1:** Summary statistics of recorded grassland aboveground biomass (kg/m^2^)

Sample number	Minimum	Maximum	Mean	Range	Standard deviation
47	1.5	4.5	3.37	3	0.7075

### High-density aboveground biomass estimation

From the original Worldview-3 image, using cross-validated data from the eight wavebands produced an *R*^2^ of 0.61 and an RMSE of 0.498 kg/m^2^ (Table 2). When using vegetation indices only, the model estimates marginally improved (*R*^2^ = 0.62, RMSE = 0.481 kg/m^2^). The standard NDVI equation performed poorly on its own, with an *R*^2^ of 0.42 and a RMSE of 0.545 kg/m^2^. This result demonstrates that the standard NIR/red band ratio computed from the original image was poorly correlated to biomass.

**Table 2:** Summary of high-density biomass model performance for each spectral variable, computed before and after image manipulation

		All variables	Bands only	All vegetation indices	Standard NDVI	Optimal model (computed from the three most important variables)
Original image	*R*^2^	0.63	0.61	0.62	0.42	0.657
	RMSE	0.479	0.498	0.481	0.545	0.420
Singular Value Decomposition and linear stretch	*R*^2^	0.71	0.67	0.69	0.32	0.713
	RMSE	0.455	0.458	0.463	0.596	0.408
Singular Value Decomposition and histogram equalization	*R*^2^	0.66	0.675	0.65	0.27	0.566
	RMSE	0.466	0.483	0.468	0.609	0.507

**Fig. 3:**
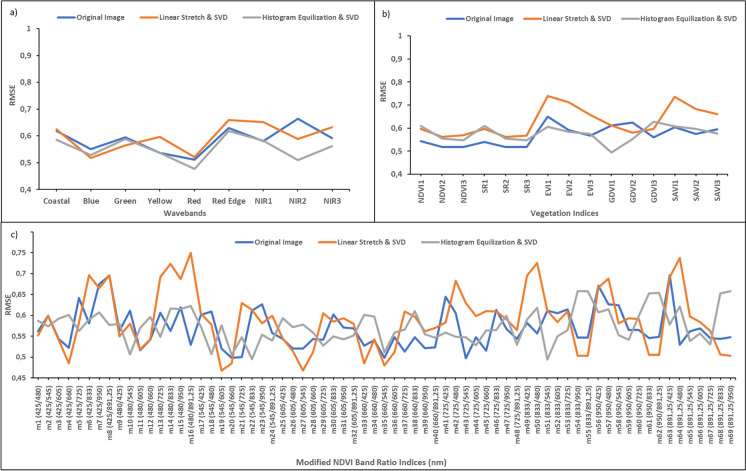
The performance of the high-density biomass estimation model with **a** Worldview-3 wavebands, **b** standard vegetation indices, and **c** modified NDVI-type indices computed from various combinations of the Worldview-3 wavebands presented in panel **a**. These were computed both before and after image manipulation (contrast enhancement and Singular Value Decomposition)

After the application of a contrast enhancement and Singular Value Decomposition (SVD), there was an improvement in biomass prediction accuracy. For instance, high-density AGB model accuracy derived from the Worldview-3 bands increased from *R*^2^ = 0.61 (for the original image) to *R*^2^ = 0.67 (for the linearly stretched image with SVD), to an *R*^2^ value of 0.675 (for the SVD and histogram equalized image) (Table 2). Whilst histogram equalization and SVD improved model accuracies for commonly used wavebands over the standard image (Fig. 3a). RMSE values for the red, Red-edge, and near-infrared regions changed from 0.51 kg/m^2^ (red), 0.62 kg/m^2^ (Red-edge), 0.58 kg/m^2^ (NIR 1), 0.66 kg/m^2^ (NIR 2), and 0.59 kg/m^2^ (NIR 3) to 0.47 kg/m^2^ (red), 0.61 kg/m^2^ (Red-edge), 0.58 kg/m^2^ (NIR 1), 0.50 kg/m^2^ (NIR 2), and 0.56 kg/m^2^ (NIR 3), respectfully (Fig. 3a).

Vegetation indices computed from the linearly stretched image and SVD improved model accuracy over the original image, with *R*^2^ values improving from 0.62 to 0.69, respectively (Table 2). Subsequently, the best performing standard vegetation indices were identified as follows: GDVI 1 (RMSE = 0.49 kg/m^2^), computed from the histogram equalization and SVD; SR 2 (RMSE = 0.51 kg/m^2^), computed from the original image; and NDVI 2 (RMSE = 0.51 kg/m^2^), computed from the original image (Fig. 3b). However, the standard NDVI equation did not show an increase in model accuracy with the use of either image enhancement technique or SVD (Table 2). The use of modified NDVI indices demonstrated an improved individual model performance over the standard vegetation indices. For the original image, average RMSE values for individual vegetation indices decreased from 0.60 kg/m^2^ (standard vegetation indices) to 0.56 kg/m^2^ (modified NDVI indices). Whilst for the linearly stretch image and SVD, average RMSE values decreased from 0.66 kg/m^2^ (standard vegetation indices) to 0.57 kg/m^2^ (modified NDVI indices), respectfully (Fig. 3b and c).

**Fig. 4:**
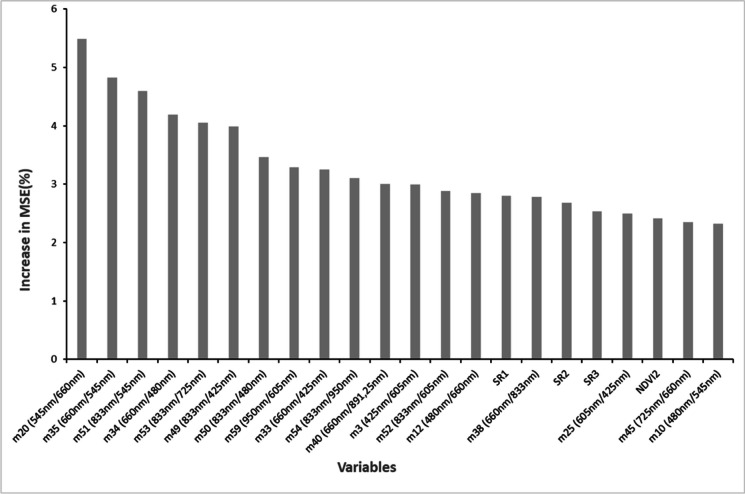
Variable importance, where a higher mean square error (MSE) represents greater importance

**Fig. 5:**
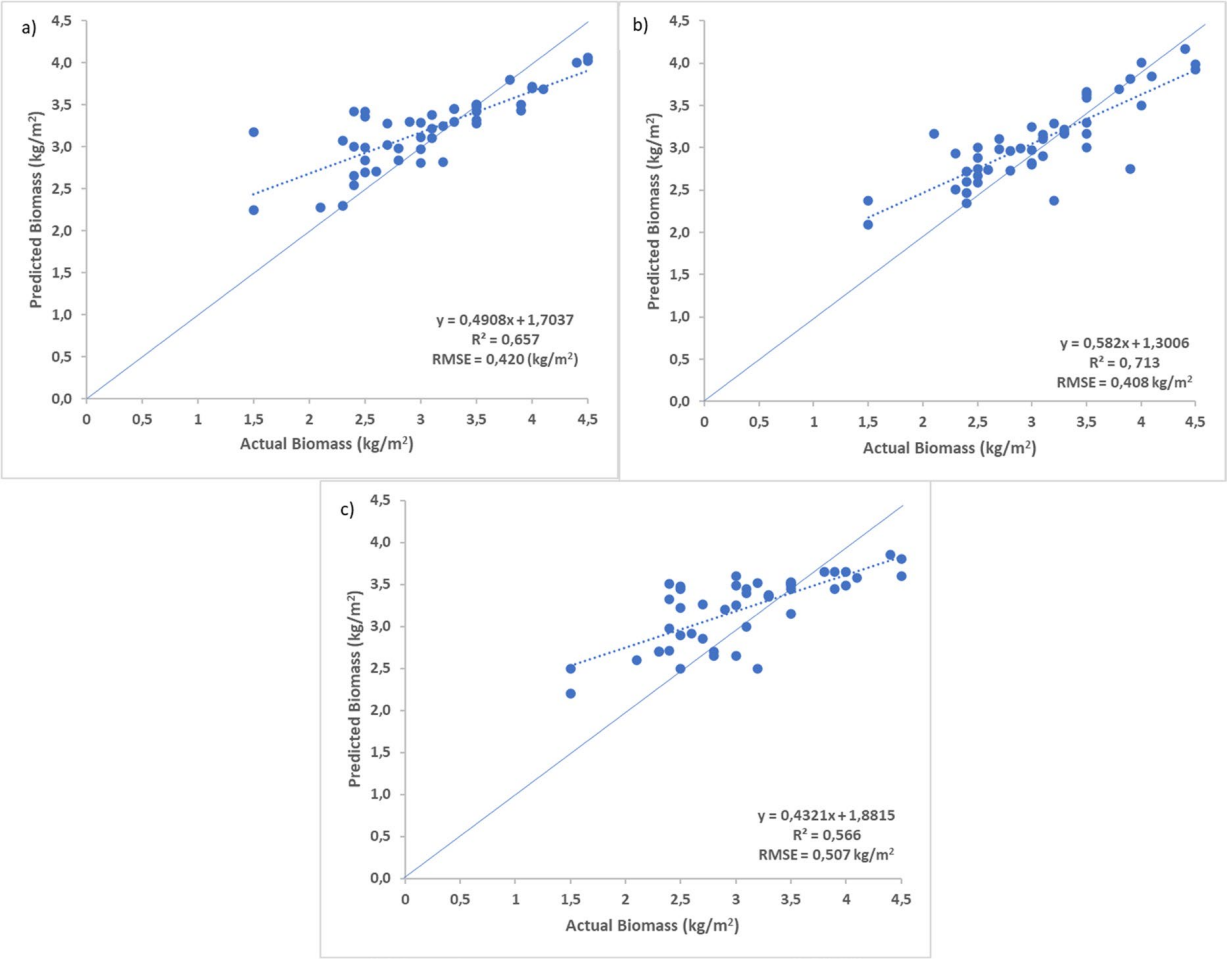
Optimal predicted vs observed biomass values within high-density biomass plots computed from the three most important variables (m20 (green_(545 nm)_ and red_(660 nm)_), m35 (red_(660 nm)_ and green_(545 nm)_), m51 (NIR 1_(833 nm)_ and green_(545 nm)_)); **a** the original image, **b** a linearly stretched image (min = 0, max = 255) and Singular Value Decomposition, and **c** an image with a histogram equalization stretch (min = 0, max = 255) and Singular Value Decomposition. The dotted line represents the line of best fit, whilst the solid blue line depicts the 1:1 line

Thereafter, 22 important variables were identified through backwards feature elimination. The most important variables identified were m20 (green_(545 nm)_ and red_(660 nm)_), m35 (red_(660 nm)_ and green_(545 nm)_), m51 (NIR 1_(833 nm)_ and green_(545 nm)_), m34 (red_(660 nm)_ and blue_(480 nm)_), and m53 (NIR 1_(833 nm)_ and Red-edge_(725 nm)_) (Fig. 4). Afterwards, the three best performing variables (m20 (green_(545 nm)_ and red_(660 nm)_), m35 (red_(660 nm)_ and green_(545 nm)_), m51 (NIR 1_(833 nm)_ and green_(545 nm)_)) were then used to compute the optimal high-density AGB models for each image. Subsequently, the linearly stretched image and SVD produced the best performing high-density AGB model, with an *R*^2^ of 0.713 and an RMSE of 0.40 kg/m^2^ (Fig. 5b).

## Discussion

The saturation of broad-band vegetation indices is a common problem within high-density biomass regions (Mutanga et al., 2012). Naturally, as biomass levels increase throughout the grass growing season, the red waveband, which contains chlorophyll absorption features, quickly reaches peak absorption (Gitelson et al., 1996). To improve biomass estimates under these conditions, narrow-band indices developed from hyperspectral data have been applied (Mutanga & Skidmore, 2004). However, hyperspectral datasets are often hindered by costs, availability, and processing (Sibanda et al., 2015a, 2015b). In this regard, the study set out to assess the use of 2 m high-resolution 8-band Worldview-3 imagery (400–1040 nm) in conjunction with modified NDVI indices and image manipulation techniques to formulate a framework to diminish the effects of biomass signal saturation within a heterogeneous tropical grassland environment. Results produced by this study demonstrated that contrast enhancement techniques combined with Singular Value Decomposition (SVD) improved high-density biomass estimates. Moreover, the study demonstrated that the effects of biomass saturation were effectively reduced through the combined use of Worldview-3 imagery, SVD, contrast stretching, and modified NDVI indices.

### The relationship between spectral variables and high-density aboveground biomass estimation

The variation in results produced by each set of input variables (i.e., wavebands only, standard vegetation indices only, modified NDVI indices only, and a combination of all the variables) confirmed the influence of signal saturation and the difficulties associated with accurately estimating high-density biomass. The Worldview-3 spectral bands alone demonstrated a moderate AGB model performance (RMSE = 0.498 kg/m^2^), whilst vegetation indices were found to have marginally improved results (RMSE = 0.481 kg/m^2^). Nonetheless, the standard NDVI index computed from the NIR/red band ratio exhibited a poor correlation to high-density AGB (*R*^2^ = 0.42). This outcome demonstrates the problem of biomass signal saturation, as the red band (630–690 nm) within the ratio rapidly saturates and constrains ratio values. Both Mutanga and Skidmore (2004) and Mutanga et al. (2012) obtained comparable results, with the standard NDVI performing poorly in estimating biomass within high-density environments. More precisely, Mutanga and Skidmore (2004) recorded an accuracy of *R*^2^ = 0.26 when using NDVI to estimate pasture biomass. Whilst Mutanga et al. (2012) noted an accuracy of *R*^2^ = 0.39 when they applied NDVI to predict wetland biomass.

Nevertheless, to improve high-density AGB estimation, variable selection identified that modified NDVI indices computed from the red (630–690 nm), green (510–580 nm), NIR (770–895 nm), and Red-edge (705–745 nm) regions were the most influential. The combination of these ratio indices proved to be significantly correlated to high-density AGB (*R*^2^ = 0.71). As discussed by Gitelson et al. (2003), healthy vegetation contains larger amounts of chloroplasts. During photosynthesis, these chloroplasts absorb greater amounts of red and blue wavelengths of light whilst reflecting near-infrared light away, which in turn facilitates a greater discernment of vegetation condition (Gitelson et al., 2003). Similarly, both Todd et al. (1998) and Thenkabail et al. (2004) found the red waveband to be correlated to biomass (*R*^2^ ≥ 0.50) within their investigations. However, in high-density environments, the amount of red light was absorbed by chloroplasts peaks — which reduces biomass sensitivity.

Consequently, the use of other visible wavebands where the difference between the two bands within the equation is not substantial may provide a solution to this issue. In particular, the green waveband (510–580 nm) due to the unique spectral peak emanating from the reflectance profile of healthy vegetation may be more effective in high-density AGB estimation (Curran, 1989). For instance, Gitelson et al. (1996) noted that indices computed from the green waveband were more receptive to increased chlorophyll-α concentrations than the standard NDVI equation — where the red band is known to rapidly saturate. In this study, it is perceived that the receptiveness of the green waveband to increased chlorophyll concentrations facilitated high-density biomass estimates.

Moreover, the Red-edge (705–745 nm) and the NIR 1 (770–895 nm) bands are particularly sensitive to instances of healthy vegetation due to increased chlorophyll content and improved leaf structure — resulting in higher spectral reflectance signals within high-density environments (Immitzer et al., 2012). The NIR 1 (770–895 nm) band is particularly useful for vegetation analysis and high-density biomass investigations due to its decreased sensitivity to atmospheric influences (Immitzer et al., 2012). These results correspond to those generated by Clevers et al. (2007), who documented both the NIR (either 777 to 858 nm or 859 to 1006 nm) and the Red-edge region (668 to 776 nm) as important for predicting fresh and dry biomass. In addition, several other studies have demonstrated the ability of both bands in improving biomass predictive capacity (Clevers et al., 2007; Karlson et al., 2015; Ramoelo et al., 2015a, 2015b; Sibanda et al., 2017). Whilst in high-density biomass conditions, both Mutanga and Skidmore (2004) and Mutanga et al. (2012) found modified band ratios derived from the Red-edge shoulder to be vital in offsetting biomass saturation. The use of broad-band modified NDVI-type indices computed from these important variables vastly improved biomass estimates within a high-density environment. This demonstrates the practical application of Worldview-3 imagery and modified NDVI indices in detecting subtle changes in vegetation condition within high-density rangelands, thereby providing a platform to assist rangeland management.

### The performance of image manipulation techniques in high-density aboveground biomass estimation

The standard NDVI equation did not demonstrate any improvement in model prediction. Although unexpected, this outcome was caused by the technique’s failure to sufficiently enhance NIR values within the standard NDVI equation. Fundamentally, as Mutanga and Skidmore (2004) observed, as vegetation coverage approaches 100%, the absorption of red light peaks, whilst NIR reflectance continues to rise due to scattering effects caused by the leaf canopy. This triggers an imbalance in red and NIR reflectance, which in turn hampers the NDVI ratio and results in a weak correlation with high-density biomass (Gitelson et al., 1996; Huete et al., 1999). As such, NIR values are required to be effectively twice that of the red values to counteract biomass saturation (Mutanga & Skidmore, 2004). Nevertheless, whilst the standard NDVI performed poorly under high-density biomass conditions, the use of contrast image enhancement techniques, single value decomposition, and modified NDVI indices developed from high-resolution Worldview-3 imagery was successful in estimating high-density biomass. Both linear contrast stretching and histogram equalization stretching enhanced the visual contrast of the Worldview-3 image by stretching the original pixel values to new minimum (0) and maximum (255) values. These dynamic changes to gray levels within the Worldview-3 image enhanced the interpretability of the image by allowing the full range of available brightness values to be considered, which facilitated SVD feature extraction (Al-amri et al., 2010; Demirel et al., 2009). Thereafter, Singular Value Decomposition improved the signal-to-noise ratio within biomass data segments comprising of laterally coherent events (signal saturation) by factoring in the greatest singular value contributions (which signify the laterally coherent signals), whilst the lowest singular values were associated with background noise (Bekara & Van der Baan, 2007). This resulted in improved high-density biomass estimates for most of the spectral variables tested. In particular, the biomass models derived from a combination of the important spectral variables and a linearly stretched image with SVD exhibited an improvement over the original image, with *R*^2^ values increasing from 0.657 to 0.713, respectively. Rulinda et al. (2012) obtained similar findings in their study of assessing vegetative drought in East Africa. Additionally, other studies have also successfully implemented both SVD and image enhancement techniques to facilitate remote sensing analysis and improve feature extraction (Bhandari et al., 2012; Demirel et al., 2009). For instance, Bhandari et al. (2012) conducted a comparative analysis of different wavelet filters — which included image equalization, Singular Value Decomposition (SVD), and discrete wavelet transformation — to enhance contrast and brightness within multispectral remote sensing images. Similarly, Demirel et al. (2009) utilized image equalization, SVD, and discrete wavelet transformation to improve satellite images. Whilst in a more applied investigation, Danaher et al. (1995) utilized SVD in combination with Landsat and SPOT imagery to effectively classify forest species (> 99% accuracy) in Wicklow County, Ireland. In a different study, Lisowski and Cook (1996) used SVD and airborne remote earth sensing (ARES) data to assist with classification analyses of the Navajo Generating Station, at Page Arizona, USA. Lastly, Marshall and Thenkabail (2015) used SVD in combination with multispectral broad-band and hyperspectral narrow-band data to successfully estimate crop biomass (*R*^2^ > 0.80).

The outcomes generated by this investigation demonstrate the feasibility of this framework to alleviate the problem of biomass saturation and monitor subtle changes in rangeland condition within high-density grassland settings.

### Limitations and opportunities for future research

This study has showcased the potential benefits of using high-resolution Worldview-3 multispectral data in conjunction with modified band ratio indices and image manipulation techniques to address the challenge of biomass signal saturation within densely vegetated grassland environments. Nonetheless, the considerable costs associated with obtaining and processing such imagery may pose specific challenges to resource-constrained stakeholders, particularly for large-scale or frequent monitoring applications (Johansen et al., 2020). In this regard, the incorporation of freely available multispectral data with coarser spatial resolutions, such as Landsat 8 and 9 or Sentinel-2, alongside modified band ratio indices and image enhancement methods, could provide unique monitoring opportunities in densely vegetated areas. Whilst coarser spatial resolution data (> 10 m) might lack the intricate details of high-resolution images, their broader coverage enables a more comprehensive understanding of extensive geographic regions, making them more suitable for regional-scale analysis (Li et al., 2021). Research by Dehghan-Shoar et al. (2023), Wang et al. (2019), Shoko et al. (2018), and Sibanda et al., (2015a, 2015b) has showcased the effectiveness of Landsat and Sentinel-2 imagery in supporting aboveground biomass (AGB) monitoring in grassland environments. Moreover, the application of modified band ratio indices and image enhancement techniques in combination with these coarser-resolution datasets could assist with accentuating specific features and thus improve data interpretability (Reddy, 2018), aiding in improved feature identification within densely vegetative areas. However, this approach necessitates careful consideration of uncertainty levels and the potential for information loss due to larger pixel sizes. Therefore, finding the right balance between spatial resolution and geographical coverage is crucial and should be tailored for specific applications. Thus, in order to gain a comprehensive grasp of how freely accessible medium resolution multispectral sensors can be effectively integrated into the framework established by this study, additional research in this domain is essential.

In addition, the imminent emergence of upcoming multispectral data sensors, such as the next generation Landsat sensor and the Worldview Legion satellite constellation (Maxar, 2020; NASA, 2021), introduces a realm of exciting possibilities for the assessment of aboveground biomass across expansive spatial extents and temporal periods. These upcoming sensors are anticipated to deliver improved spatial resolutions (0.29 m via Worldview Legion and 10 m through Landsat NeXt), heightened spectral sensitivity (featuring 9 spectral bands with Worldview Legion and 26 spectral bands with Landsat NeXt), and more frequent revisits (hourly revisits with Worldview Legion and a revisit interval of 6 days with Landsat NeXt) (Maxar, 2020; NASA, 2021; Taylor, 2022). Consequently, with a focused emphasis on their finer spectral bands, these sensors have the potential to capture a broader spectrum of biomass variations, differentiating densely vegetated areas in far greater detail. Additionally, the augmented revisit frequencies associated with these platforms will enable more frequent data collection, facilitating temporal analysis and mitigating data gaps in dynamic landscapes. Furthermore, the enhanced spatial resolution may enable the characterization of smaller-scale features and more accurate mapping of heterogeneous landscapes, crucial for understanding ecosystem variability. The combined benefits of improved spectral and spatial configurations in upcoming multispectral sensors pave the way for more reliable and comprehensive biomass estimations (Wulder et al., 2022). Thus, with access to these enhanced capabilities, researchers and practitioners can gain a more comprehensive understanding of regional and global AGB stocks, contributing to better-informed decision-making for ecosystem management and climate change mitigation. However, the successful assimilation of these advanced datasets calls for a concerted effort in addressing processing intricacies, interoperability challenges, and integration complexities within existing methodological frameworks. This underscores the importance of continual research and collaboration within the remote sensing community to harness the full potential of advanced modern multispectral data sources for effective AGB estimation and environmental monitoring.

Nevertheless, although the study has presented a viable strategy to counter the impact of biomass signal saturation within densely vegetated areas, it is imperative for forthcoming research to explore alternative methodologies to improve model accuracies and reduce data complexities. To this end, future research should explore the feasibility of the Wide Dynamic Range Vegetation Index (WDRVI) developed by Gitelson (2004) in combination with modern and next generation satellite sensors and image enhancement techniques as an alternative approach. The WDRVI, which is an extension of the widely utilized NDVI, offers the flexibility to downweigh NIR reflectance using a customizable constant, tailored to specific vegetation canopies (Aguilar-Amuchastegui and Henebry 2008; Viña & Gitelson, 2005). Through this parameter adjustment, the WDRVI facilitates precise calibration of biomass estimation in regions where conventional NDVI indices might encounter saturation (Gitelson, 2004; Vina et al., 2004). Furthermore, this approach presents a potential alternative for AGB estimation in densely vegetated regions, where capturing subtle changes in vegetation dynamics and biomass fluctuations is essential for effective carbon monitoring and ecosystem management. 

## Conclusion

This study sought to evaluate the use of high-resolution Worldview-3 imagery in conjunction with modified band ratio indices and image manipulation techniques to reduce the effects of biomass signal saturation within a complex tropical grassland environment. Based on the results of this study, our principal conclusion is that the effect of biomass saturation was effectively removed through the combined use of Worldview-3 imagery, linear contrast stretching, Singular Value Decomposition, and modified NDVI indices. More specifically, it was discovered that modified NDVI indices developed from the red, green, and near-infrared bands facilitated greater high-density grassland biomass estimates. Whilst the findings generated from this study provides a platform for rangeland managers to regionally assess slight changes in vegetation condition within high-density South African grasslands, there remains room for improvement. As such, the development of more robust algorithms combined with additional remotely sensed or topographical and environmental datasets may enable future research to better detect subtle changes in vegetation condition within high-density environments.

## Data Availability

Data are available from the authors upon request.

## Appendix 1. Description of modified NDVI vegetation indices

**Table:**

Modified vegetation index	Band combination
m1	Coastal (425 nm) & blue (480 nm)
m2	Coastal (425 nm) & green (545 nm)
m3	Coastal (425 nm) & yellow (605 nm)
m4	Coastal (425 nm) & red (660 nm)
m5	Coastal (425 nm) & Red-edge (725 nm)
m6	Coastal (425 nm) & NIR1 (833 nm)
m7	Coastal (425 nm) & NIR2 (950 nm)
m8	Coastal (425 nm) & NIR3 (891.25 nm)
m9	Blue (480 nm) & Coastal (425 nm)
m10	Blue (480 nm) & green (545 nm)
m11	Blue (480 nm) & yellow (605 nm)
m12	Blue (480 nm) & red (660 nm)
m13	Blue (480 nm) & Red-edge (725 nm)
m14	Blue (480 nm) & NIR1 (833 nm)
m15	Blue (480 nm) & NIR2 (950 nm)
m16	Blue (480 nm) & NIR3 (891.25 nm)
m17	Green (545 nm) & Coastal (425 nm)
m18	Green (545 nm) & blue (480 nm)
m19	Green (545 nm) & yellow (605 nm)
m20	Green (545 nm) & red (660 nm)
m21	Green (545 nm) & Red-edge (725 nm)
m22	Green (545 nm) & NIR 1 (833 nm)
m23	Green (545 nm) & NIR2 (950 nm)
m24	Green (545 nm) & NIR3 (891.25 nm)
m25	Yellow (605 nm) & Coastal (425 nm)
m26	Yellow (605 nm) & blue (480 nm)
m27	Yellow (605 nm) & green (545 nm)
m28	Yellow (605 nm) & red (660 nm)
m29	Yellow (605 nm) & Red-edge (725 nm)
m30	Yellow (605 nm) & NIR1 (833 nm)
m31	Yellow (605 nm) & NIR2 (950 nm)
m32	Yellow (605 nm) & NIR3 (891.25 nm)
m33	Red (660 nm) & Coastal (425 nm)
m34	Red (660 nm) & blue (480 nm)
m35	Red (660 nm) & green (545 nm)
m36	Red (660 nm) & yellow (605 nm)
m37	Red (660 nm) & Red-edge (725 nm)
m38	Red (660 nm) & NIR1 (833 nm)
m39	Red (660 nm) & NIR2 (950 nm)
m40	Red (660 nm) & NIR3 (891.25 nm)
m41	Red-edge (725 nm) & Coastal (425 nm)
m42	Red-edge (725 nm) & blue (480 nm)
m43	Red-edge (725 nm) & green (545 nm)
m44	Red-edge (725 nm) & yellow (605 nm)
m45	Red-edge (725 nm) & red (660 nm)
m46	Red-edge (725 nm) & NIR1 (833 nm)
m47	Red-edge (725 nm) & NIR2 (950 nm)
m48	Red-edge (725 nm) & NIR3 (891.25 nm)
m49	NIR1 (833 nm) & Coastal (425 nm)
m50	NIR1 (833 nm) & blue (480 nm)
m51	NIR1 (833 nm) & green (545 nm)
m52	NIR1 (833 nm) & yellow (605 nm)
m53	NIR1 (833 nm) & Red-edge (725 nm)
m54	NIR1 (833 nm) & NIR2 (950 nm)
m55	NIR1 (833 nm) & NIR3 (891.25 nm)
m56	NIR2 (950 nm) & Coastal (425 nm)
m57	NIR2 (950 nm) & blue (480 nm)
m58	NIR2 (950 nm) & green (545 nm)
m59	NIR2 (950 nm) & yellow (605 nm)
m60	NIR2 (950 nm) & Red-edge (725 nm)
m61	NIR2 (950 nm) & NIR1 (833 nm)
m62	NIR2 (950 nm) & NIR3 (891.25 nm)
m63	NIR3 (891.25 nm) & Coastal (425 nm)
m64	NIR3 (891.25 nm) & blue (480 nm)
m65	NIR3 (891.25 nm) & green (545 nm)
m66	NIR3 (891.25 nm) & yellow (605 nm)
m67	NIR3 (891.25 nm) & Red-edge (725 nm)
m68	NIR3 (891.25 nm) & NIR1 (833 nm)
m69	NIR3 (891.25 nm) & NIR2 (950 nm)

## Appendix 2. Description of traditional vegetation indices and formulas used

**Table:**

Vegetation index	Formula	Reference
Normalized difference vegetation index (NDVI)	(NIR1 − red)/(NIR1 + red)	Rouse et al. (1974)
Normalized difference vegetation index (NDVI2)	(NIR2 − red)/(NIR2 + red)	Rouse et al. (1974)
Normalized difference vegetation index (NDVI3)	(NIR3 − red)/(NIR3 + red)	Rouse et al. (1974)
Simple ratio (SR)	NIR1/red	Birth and McVey (1968)
Simple ratio (SR2)	NIR2/red	Birth and McVey (1968)
Simple ratio (SR3)	NIR3/red	Birth and McVey (1968)
Enhanced vegetation index (EVI)	EVI = 2.5 × [(NIR1 − red)/(NIR1 + (6 × red − 7.5 × blue) + 1)]	Huete et al. (2002)
Enhanced vegetation index (EVI2)	EVI = 2.5 × [(NIR2 − red)/(NIR2 + (6 × red − 7.5 × blue) + 1)]	Huete et al. (2002)
Enhanced vegetation index (EVI3)	EVI = 2.5 × [(NIR3 − red)/(NIR3 + (6 × red − 7.5 × blue) + 1)]	Huete et al. (2002)
Green difference vegetation index (GDVI)	(NIR1 − green)/(NIR1 + green)	Sripada et al. (2006)
Green difference vegetation index (GDVI2)	(NIR2 − green)/(NIR2 + green)	Sripada et al. (2006)
Green difference vegetation index (GDVI3)	(NIR3 − green)/(NIR3 + green)	Sripada et al. (2006)
Soil-adjusted vegetation index (SAVI)	[(NIR – red)/(NIR + red + *L*)] × (1 + *L*) (where *L* = 0.5)	Huete (1988)
Soil-adjusted vegetation index (SAVI2)	[(NIR2 – red)/(NIR2 + red + *L*)] × (1 + *L*) (where *L* = 0.5)	Huete (1988)
Soil-adjusted vegetation index (SAVI3)	[(NIR3 – red)/(NIR3 + red + *L*)] × (1 + *L*) (where *L* = 0.5)	Huete (1988)
